# Universal Confinement Length Governs Radical Depolymerization Kinetics in Nanoconfined Polymers

**DOI:** 10.1002/smll.75295

**Published:** 2026-08-18

**Authors:** Uiseok Hwang, Ching‐Yu Wang, Zhanyuan Liu, John M. Vohs, Baekmin Q. Kim, Daeyeon Lee

**Affiliations:** ^1^ Department of Chemical and Biomolecular Engineering School of Engineering and Applied Science University of Pennsylvania Philadelphia Pennsylvania USA; ^2^ Department of Battery and Chemical Engineering Hanyang University ERICA Ansan Gyeonggi Republic of Korea

**Keywords:** nanoconfinement, polymer nanocomposites, polymer upcycling, thermal depolymerization

## Abstract

Thermal depolymerization is typically studied in bulk, yet many systems involve polymers confined within nanoscale geometries. Understanding how nanoconfinement alters radical depolymerization kinetics is essential for interpreting polymer stability in nanostructured environments. Here we show that thermal depolymerization of poly(methyl methacrylate) (PMMA) is governed by a universal confinement length scale that suppresses radical unzipping and increases the onset depolymerization temperature by up to ∼150°C. We systematically introduce confinement in two geometries: 1D thin films and 3D interstitial pores within SiO_2_ nanoparticle packings. Nanoconfinement progressively suppresses chain‐end‐initiated unzipping and shifts the onset of random‐chain‐scission‐initiated depolymerization to higher temperatures. This behavior stems from restricted segmental mobility near polymer–SiO_2_ interfaces, imposing kinetic constraints on radical propagation. By defining an effective confinement length unifying 1D and 3D architectures, data from both geometries collapse onto a single master curve, revealing a critical threshold at ∼7.2 *R*_g_ of the PMMA chains. Below this scale, depolymerization enters a confinement‐saturated regime where further dimensional reduction no longer increases the depolymerization temperature. This collapse demonstrates that depolymerization kinetics are governed by a universal length scale independent of specific architecture, establishing nanoconfinement as a fundamental physical parameter governing radical reaction dynamics in nanostructured environments.

## Graphical abstract

Analysis of PMMA depolymerization kinetics identifies a critical threshold under extreme nanoconfinement where segmental mobility saturation prevents further increases in depolymerization temperature.

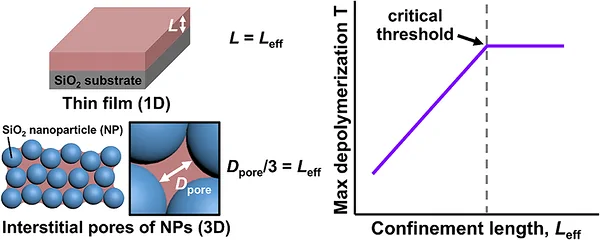

## 1 Introduction

Thermal depolymerization is widely explored as a route for recovering monomers from commodity polymers and enabling a circular polymer economy [1, 2, 3, 4, 5]. While the intrinsic chemical mechanisms governing radical depolymerization are well established [5, 6, 7, 8, 9, 10, 11], far less is understood about how the physical environment surrounding the polymer influences these reactions. In many practical systems, polymers are not present as bulk melts but instead reside within confined geometries such as thin films [12, 13], nanoporous catalysts [14, 15], or polymer–nanoparticle (NP) composites [16, 17, 18]. Under such conditions, spatial confinement and interfacial interactions can significantly modify polymer chain mobility and the resulting reaction dynamics. Understanding how nanoconfinement influences depolymerization kinetics is therefore essential for interpreting polymer stability and degradation in nanostructured environments.

When polymers are confined within nanoscopic spaces such as thin films on substrates or within nanopores, their physical properties and chain conformations are significantly modified. These changes can lead to deviations in glass transition temperature (T_g_), segmental dynamics, viscoelastic response, and physical aging relative to bulk, owing to spatial constraints and interfacial interactions [19, 20, 21, 22, 23, 24, 25, 26, 27]. Such nanoconfinement effects can also influence chemical reactions, as exemplified by altered degradation behavior in confined polymers [4, 28, 29, 30, 31]. Beyond these classical observations, recent studies have highlighted nanoconfinement as a fundamental regulator of molecular assembly and structural ordering at the air–water interface [32, 33, 34, 35]. Additionally, nanoconfinement is particularly relevant in catalytic thermal deconstruction and depolymerization that involve polymer chain infiltration or adsorption within the nanoporous channels of solid catalysts [36, 37]. While these systems are designed to lower activation energy barriers via chemical interactions, accompanying confinement may introduce competing kinetic limitations, such as reduced chain mobility and transport restrictions of reactive chain ends or volatile products [36].

Despite extensive studies on polymer degradation and depolymerization, it remains unclear whether nanoscale confinement introduces a general physical framework governing radical depolymerization kinetics. It is unknown whether depolymerization behavior in distinct confinement geometries such as thin films and nanoporous environments can be described using a common length scale that captures the influence of interfacial interactions and restricted chain mobility. Establishing such a framework would provide a unified basis for interpreting polymer stability and reaction dynamics across a wide range of nanostructured materials. Here we show that radical depolymerization kinetics are governed by a universal confinement length scale that unifies thin‐film and nanoporous confinement environments.

To elucidate how nanoconfinement influences radical depolymerization kinetics, we investigate the thermal depolymerization behavior of poly(methyl methacrylate) (PMMA) under systematically controlled confinement environments. Confinement is introduced using SiO_2_ surfaces in two distinct geometries: 1D confined thin films on flat SiO_2_ substrates and 3D confinement within the interstitial voids of disordered SiO_2_ NP packings, achieved via the solvent‐driven infiltration of polymer (SIP) technique [38, 39, 40, 41]. The degree of confinement is systematically controlled by varying the thickness of PMMA thin films (1D confinement) and NP diameters that define the interstitial void dimensions (3D confinement). To reconcile these different architectures, we define an effective confinement length (*L*_eff_) based on the volume‐to‐surface ratio, allowing for unified comparison of 1D and 3D confinement environments. Our observations suggest that, as the degree of confinement increases, end‐group‐initiated depolymerization is progressively suppressed, while the onset temperature associated with random‐chain‐scission‐initiated depolymerization shifts to higher values, indicating enhanced thermal stability. A distinct transition in thermal behavior is observed at a critical threshold of *L*_eff_ ∼60 nm, corresponding to ∼7.2 times the radius of gyration (*R*_g_) of the polymer chain. This length scale is consistent with prior reports indicating that the transition from bulk‐like to confinement‐dominated behavior occurs at confinement dimensions of ∼7 *R*_g_ [42, 43]. Below this length scale (*L*_eff_ ∼60 nm), depolymerization enters a confinement‐saturated regime in which further reductions in spatial dimension no longer increase the depolymerization temperature. This framework allows depolymerization kinetics from distinct confinement geometries to be compared on a common physical basis. These findings establish a quantitative framework for understanding the thermal depolymerization of PMMA under nanoconfinement and identify a critical threshold for spatial constraints, offering essential insights for the rational design and optimization of depolymerization systems, as well as for the development of thermally stable polymer nanocomposites.

## 2 Results and Discussion

We first establish the bulk depolymerization mechanism of PMMA before examining how nanoconfinement alters the relative contributions of the two radical initiation pathways. We employ a structurally well‐defined, commercially available poly(methyl methacrylate) (PMMA) synthesized via anionic polymerization, with a number‐average molecular weight (*M*_n_) of 99 kg mol^−1^ and a polydispersity index (PDI) of 1.12 (Figure 1a). The PMMA chains possess a diphenyl methyl end‐group (according to the manufacturer) that is thermally more labile than the polymer backbone. The narrow molecular weight distribution and well‐defined molecular structure enable systematic investigation of nanoconfinement effects on thermal depolymerization while minimizing the influence of structural heterogeneity. We monitor real‐time depolymerization kinetics using thermogravimetric analysis coupled with mass spectrometry (TGA–MS) under vacuum. During heating, thermal energy supplied in the furnace induces chemical depolymerization to volatile methyl methacrylate (MMA) monomers, whereas subsequent fragmentation into diagnostic ions (e.g., m/z = 41) occurs during electron‐impact ionization in the MS chamber, as schematically illustrated in Figure 1a.

**FIGURE 1:**
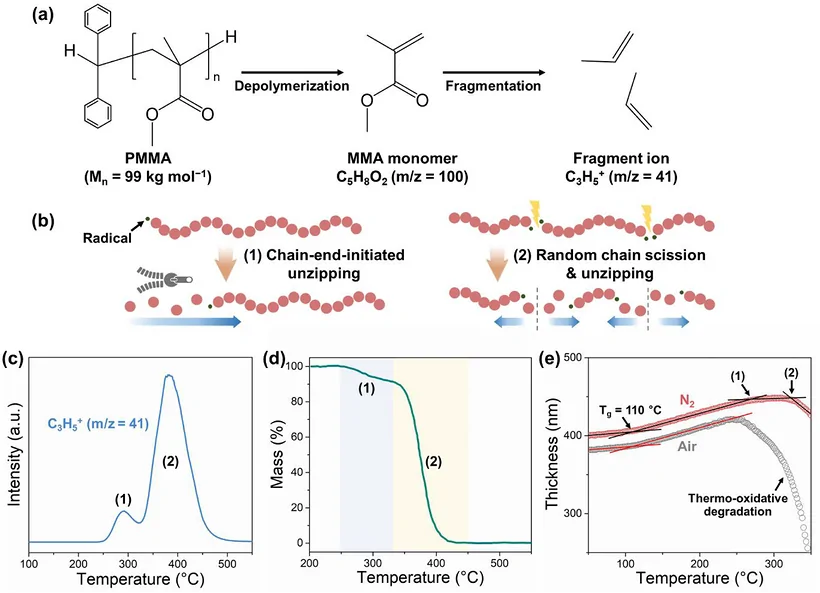
(a) Schematic illustration of thermal depolymerization of PMMA and subsequent diagnostic ion fragmentation during TGA–MS measurements. (b) Two distinct thermal depolymerization pathways of PMMA: (1) chain‐end‐initiated unzipping and (2) random chain scission followed by unzipping. (c) Temperature‐dependent ion evolution profile of PMMA powder showing the representative fragment ion C_3_H_5_^+^ (m/z = 41). (d) Corresponding TGA mass‐loss curve of PMMA powder obtained during TGA–MS analysis. (e) Temperature‐dependent thickness profiles of PMMA films on silanized Si substrates measured under ambient and nitrogen atmospheres.

The thermal depolymerization of PMMA proceeds via a radical unzipping mechanism. Radical generation occurs through two distinct initiation pathways (Figure 1b): (1) thermal cleavage of labile end groups leading to activation of chain ends, and (2) random chain scission via homolytic C─C bond cleavage along the polymer backbone [44, 45, 46]. In both cases, the resulting chain‐end radical propagates through rapid unzipping, sequentially releasing MMA monomers until termination occurs. These two initiation pathways are expected to yield distinct molecular‐weight signatures during depolymerization: chain‐end‐initiated unzipping decreases molecular weight gradually, whereas random‐chain‐scission‐initiated depolymerization reduces it sharply even at low conversion. Because cleavage of labile end groups requires lower activation energy than backbone scission, the chain‐end‐initiated pathway typically precedes the random‐chain‐scission‐initiated pathway during thermal ramping [47]. The combined contributions of these pathways determine the overall ion evolution profile, where the specific characteristics of the dominant initiation mechanism are reflected in the onset and peak temperatures of monomer evolution.

For the detection of volatilized MMA monomers by MS, the representative fragment C_3_H_5_^+^ (m/z = 41) is monitored, which arises from electron‐impact fragmentation of MMA (additional fragments are detailed in Figure S1) [48, 49]. As shown in Figure 1c, the temperature‐dependent ion evolution profiles of the PMMA powder exhibit a clear two‐step depolymerization behavior, characterized by two distinct peaks centered at ∼290°C and ∼380°C. The first peak at ∼290°C is attributed to unzipping initiated from pre‐existing chain‐end groups, whereas the second, more prominent peak at ∼380°C corresponds to depolymerization triggered by random chain scission of the polymer backbone [46]. The close correspondence among all monitored m/z signals indicates that these fragments originate from a common monomeric source released during thermal depolymerization. The TGA mass‐loss profile in Figure 1d shows good agreement with the ion evolution profiles, further supporting the two‐step depolymerization process. The first mass loss step of ∼8% closely matches the integrated area of the first MS peak (8.7%) in the m/z = 41 evolution profile, confirming its assignment to chain‐end‐initiated unzipping (Figure S2).

Investigating the temperature‐dependent thickness evolution of thin films prepared from the PMMA powder provides further support for our understanding of the depolymerization pathways derived from TGA–MS measurements. We employ in situ ellipsometry to track the thickness of PMMA films over a range of temperatures under ambient and nitrogen atmospheres; the nitrogen atmosphere approximates the vacuum conditions used in TGA–MS measurements. The films are prepared on Si substrates whose surfaces are modified with (3‐glycidoxypropyl)triethoxysilane (GPTES), which converts reactive silanol groups into epoxy groups that do not engage in hydrogen bonding, to minimize PMMA–SiO_2_ interfacial interactions [50]. As shown in Figure 1e, a distinct change in the thermal expansion slope is observed at ∼110°C in both environments, corresponding to the T_g_ of PMMA [21, 22]. Under nitrogen, following the glass transition, the thickness profiles exhibit two successive downward inflections, deviating from the linear expansion regime. This feature is consistent with the onset of the two‐step depolymerization process: the first downward deviation likely corresponds to initiation of chain‐end‐initiated unzipping at relatively lower temperatures (∼260°C), whereas the second, steeper decline indicates the onset of random‐chain‐scission‐initiated unzipping at higher temperatures (∼320°C). At 320°C, the difference between the measured thickness and the value extrapolated from the thermal expansion slope is ∼25 nm, corresponding to a 5.4% reduction in thickness due to thermal depolymerization. This value is comparable to the ∼8% mass loss observed in TGA–MS measurements, indicating good agreement between the two independent measurements. Under ambient atmosphere, the two distinct inflections associated with thermal depolymerization are obscured by the onset of thermo‐oxidative degradation at ∼250°C. Unlike the clean unzipping process observed under inert conditions, oxygen facilitates the formation of peroxy radicals (ROO•) and hydroperoxide intermediates (ROOH), which trigger non‐specific oxidative scission of the polymer backbone. This pathway lowers the initial decomposition temperature and also results in the evolution of diverse oxygenated byproducts, such as CO, CO_2_, and water, thereby competing with and suppressing the systematic generation of MMA monomers [51, 52].

The role of the thermally labile end group in the low‐temperature depolymerization pathway is confirmed through controlled thermal aging experiments. To selectively remove this moiety, the PMMA powder is pre‐heated at 200°C overnight in a vacuum oven [9, 53]. At 200°C, cleavage of the thermally labile end group proceeds too slowly to produce a detectable mass‐loss signal during the relatively rapid TGA–MS heating ramp, but the prolonged overnight exposure provides sufficient time for cleavage. Using the activation energy reported for random‐chain‐scission initiation under inert conditions (233 kJ/mol) [54], the rate of backbone scission at 200°C is estimated to be several orders of magnitude slower than at the random‐chain‐scission onset temperature observed in this study (∼380°C), indicating that the impact on the backbone remains negligible during overnight pre‐heating. At 200°C, unzipping from the radical generated upon end‐group cleavage is kinetically limited, and the radical is expected to terminate after releasing only a limited number of monomer units. This provides a plausible explanation for the removal of the labile end group without appreciable change in molecular weight [54].

The ion fragment at m/z = 41 exhibits the highest peak intensity among the characteristic ions (Figure 1c) and is therefore used as a representative probe for monitoring depolymerization kinetics. As presented in Figure 2a, the first depolymerization peak corresponding to chain‐end‐initiated unzipping disappears after pre‐heating, while the remaining peak associated with random chain scission shifts to a higher temperature. The same trend is observed in the corresponding TGA mass‐loss curves (Figure 2b), where the onset of mass loss is delayed in the pre‐heated sample relative to the untreated powder. This confirms that the labile end group acts as a low‐temperature initiation trigger, and its removal shifts depolymerization onset to the higher temperature required for random chain scission. Although the precise molecular‐level mechanism cannot be established with the present techniques, the increase in the apparent activation energy for random‐chain scission after pre‐heating is consistent with the absence of continuous end‐group‐derived radical generation during subsequent heating. In the non‐pre‐heated sample, chain‐end radicals generated by cleavage of the diphenyl methyl end groups overlap with the temperature range where random‐chain scission becomes significant, so the measured degradation behavior reflects the combined contributions of end‐group‐derived radical chemistry and random‐chain scission, leading to a lower apparent activation energy.

**FIGURE 2:**
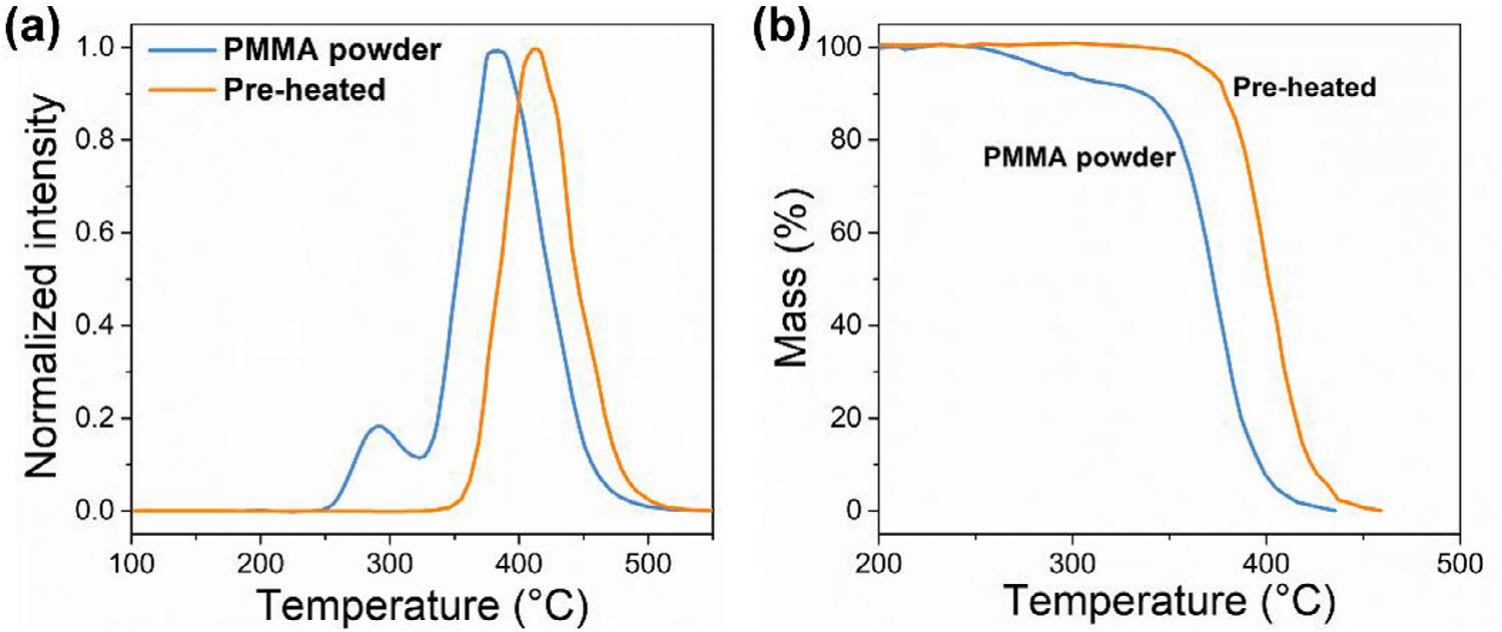
(a) Normalized temperature‐dependent ion evolution profiles (m/z = 41) and (b) corresponding TGA mass‐loss curves of the untreated and pre‐heated (200°C overnight) PMMA powders.

Building upon the established bulk depolymerization behavior of the PMMA powder, we examine deviations in depolymerization kinetics under nanoconfinement by preparing thin films (1D confinement). As shown in Figure 3a, the free‐standing film with a thickness (*L*) of 60 µm exhibits peak positions and profiles nearly identical to those of the bulk powder, indicating that, in this bulk‐like regime, the macroscopic geometry does not significantly alter the depolymerization kinetics. To elucidate the effect of confinement more systematically, we vary the thickness of PMMA films on SiO_2_ surfaces from 6 µm to 15 nm, thereby increasing the degree of confinement. As shown in Figure 3b, the ion evolution profiles reveal progressive suppression of the chain‐end‐initiated unzipping peak as the film thickness decreases, accompanied by a shift toward higher temperature. When the thickness is reduced below ∼600 nm, the low‐temperature peak associated with chain‐end‐initiated unzipping becomes indistinguishable, indicating that increasing nanoconfinement progressively suppresses this initiation pathway. Concurrently, the maximum depolymerization temperature (*T*_max_), defined as the peak temperature associated with random‐chain‐scission‐initiated unzipping, shifts progressively to higher values as the extent of confinement increases.

**FIGURE 3:**
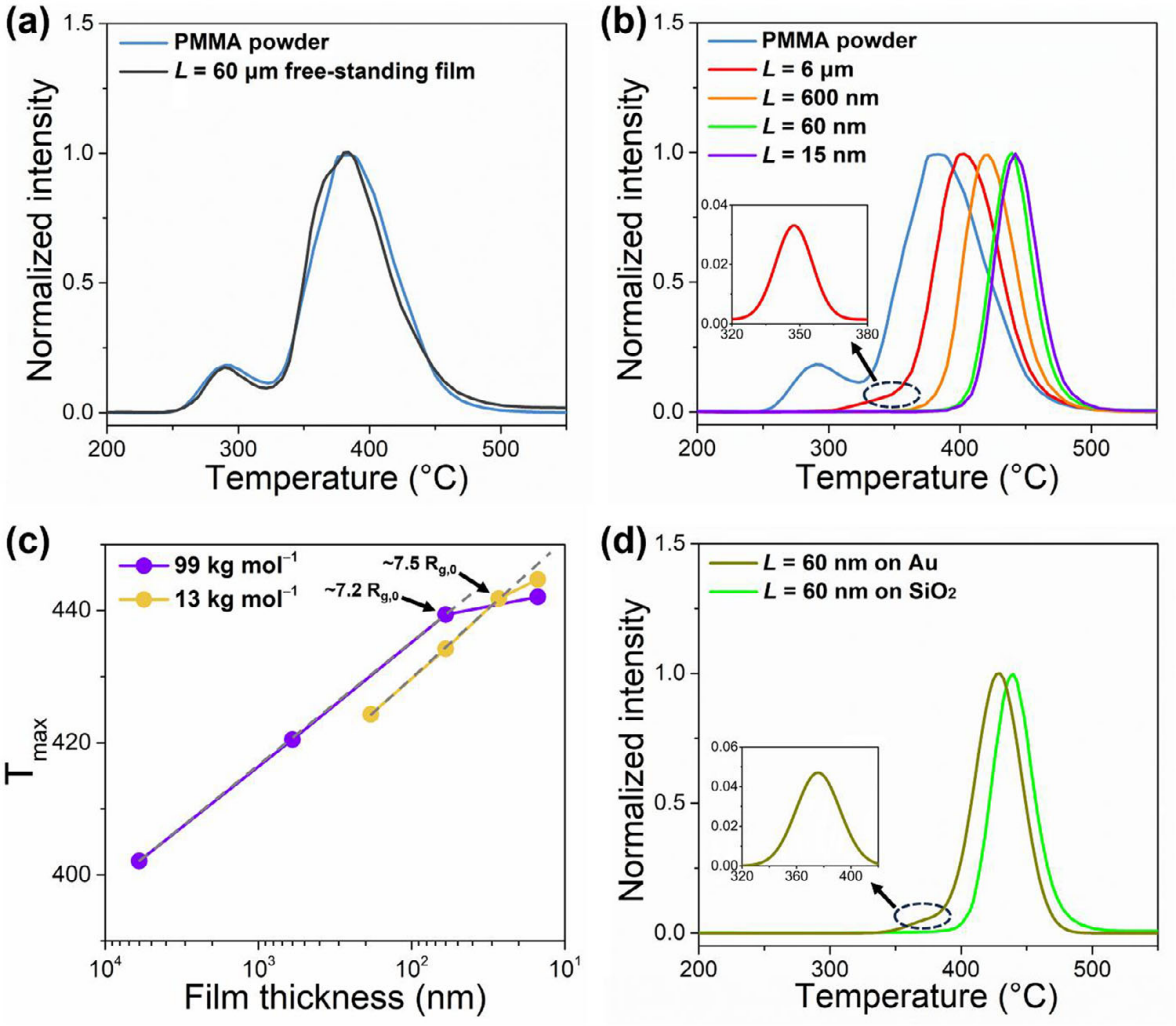
(a) Normalized temperature‐dependent ion evolution profiles (m/z = 41) of the PMMA powder and a free‐standing 60 µm‐thick PMMA film. (b) Normalized temperature‐dependent ion evolution profiles (m/z = 41) of PMMA films with varying thicknesses on SiO_2_ surfaces, together with the PMMA powder as a bulk reference. Inset: deconvoluted chain‐end‐initiated unzipping profile obtained from the fitting shown in Figure S4. (c) *T*_max_ of SiO_2_‐supported PMMA films with different molecular weights (*M*_n_ = 99 and 13 kg mol^−1^) as a function of film thickness. The *T*_max_ values exhibit a linear dependence on log *L*, following the fits *T*_max_ = −18.6 log *L* + 472.4 and *T*_max_ = −22.0 log *L* + 473.9 (indicated by gray dashed lines). Deviations from the logarithmic scaling occur at ∼60 nm (∼ 7.2 *R*_g,0_) and ∼27 nm (∼ 7.5 *R*_g,0_), respectively, indicating the onset of a confinement‐saturated regime in which further reductions in film thickness no longer increase the depolymerization temperature. (d) Normalized temperature‐dependent ion evolution profiles (m/z = 41) for 60 nm‐thick PMMA films deposited on Au and SiO_2_ surfaces, highlighting the influence of polymer–substrate interactions on depolymerization behavior. Inset: deconvoluted chain‐end‐initiated unzipping profile obtained from the fitting shown in Figure S5.

These changes in both depolymerization pathways are consistent with restricted segmental mobility near the polymer–substrate interface, likely mediated by hydrogen‐bonding interactions between the carbonyl groups of PMMA and silanol groups on the SiO_2_ surface [55]. We note that cleavage of the thermally labile end group itself is a local reaction and is therefore unlikely to be significantly affected by nanoconfinement. Although end‐group cleavage can still occur, subsequent unzipping propagation requires continuous chain motion to release monomer units sequentially. The reduced segmental mobility under confinement imposes kinetic constraints on this propagation process, thereby suppressing the chain‐end‐initiated depolymerization pathway before sufficient monomer evolution becomes detectable. This restricted chain mobility also delays random‐chain‐scission‐initiated depolymerization, contributing to the enhanced thermal stability observed under confinement [29].

As presented in Figure 3c, *T*_max_ exhibits a linear dependence on the logarithm of film thickness over the range from 6 µm down to 60 nm (gray dashed line). This logarithmic scaling indicates that interfacial constraints proportionally increase the effective activation barrier for depolymerization as spatial confinement intensifies, allowing the depolymerization temperature to be predicted from film thickness. However, the 15 nm film deviates from this linear trend, exhibiting a *T*_max_ (∼440°C) comparable to that of the 60 nm film. This deviation identifies a critical confinement length of approximately 60 nm, below which further geometric confinement no longer increases the depolymerization temperature. In this regime, nanoconfinement‐induced stabilization reaches saturation, indicating that polymer segmental mobility has already become maximally constrained by interfacial interactions. Given that the *R*_g_ of the unperturbed PMMA chains (*M*_n_ = 99 kg mol^−1^) in bulk is estimated to be ∼8.3 nm using the empirical relation *R*_g,0_ = 0.025 *M*_w_^0.5^ [56, 57], the 60 nm threshold corresponds to ∼7.2 *R*_g,0_. This scaling behavior is further validated by our observations with a lower‐molecular‐weight PMMA (*M*_n_ = 13 kg mol^−1^), which exhibits a threshold at 27 nm. This value similarly aligns with the ∼7.5 *R*_g,0_ (where *R*_g,0_ = ∼3.6 nm), demonstrating a consistent scaling relationship despite the difference in absolute chain dimensions (see Figure S3 for details). This comparison indicates that the initial molecular weight does not alter the depolymerization mechanism itself, but instead sets the absolute confinement length at which the transition to the saturated regime occurs. This result is consistent with prior literature reporting that the onset of confinement‐dominated regimes typically occurs near 7 *R*_g,0_ [42, 43]. Because this threshold substantially exceeds the dimension of an individual chain segment, the observed stabilization cannot be attributed solely to a localized interfacial effect. Rather, it suggests that confinement effects extend beyond a localized interfacial layer and involve cooperative chain dynamics over length scales larger than individual polymer segments.

To further clarify the role of PMMA–substrate interactions in the observed confinement effect on depolymerization, we employ an Au‐sputtered Si wafer as a control substrate to dramatically weaken the surface–polymer interaction. Compared to SiO_2_, Au exhibits weaker interactions with PMMA [58]. As shown in Figure 3d, for the same film thickness of 60 nm, the PMMA film on the Au surface exhibits a lower *T*_max_ (∼430°C) than that on the native SiO_2_ surface (∼440°C). Moreover, the ion evolution profile on Au reveals the reappearance of a low‐temperature peak associated with chain‐end‐initiated unzipping. This contrast suggests that stronger polymer–substrate interactions induce greater thermal stabilization under identical geometrical confinement.

We assess a confinement regime beyond that attainable in 1D thin films (15 nm) by infiltrating PMMA into densely packed SiO_2_ NP packings, thereby establishing a 3D nanoconfining environment. In these nanocomposites, polymer chains experience severe spatial confinement within the interstitial voids between adjacent NPs. The NP diameter (*D*_NP_) dictates the size of the effective interstitial pore (*D*_pore_) in random dense packing according to the geometric relationship *D*_pore_ = 0.29·*D*_NP_ [19]. This relationship has been directly confirmed for these NP packings by ellipsometric porosimetry in our previous work [59]. Accordingly, decreasing the NP diameter reduces the characteristic pore dimension accessible to the polymer, thereby increasing the degree of confinement. In this study, we employ NPs with diameters of 75, 22, and 7 nm, corresponding to well‐defined nanoconfinement environments with *D*_pore_ values of ∼21.8, ∼6.4, and ∼2.0 nm, respectively. The interstitial pores in a random dense packing of spheres are intrinsically heterogeneous in size and shape, even for monodisperse NPs, which may contribute to broadening of the depolymerization peak. We therefore use *T*_max_, which represents the dominant temperature of the convoluted depolymerization response arising from the distribution of local confinement environments, as the representative metric.

The nanocomposite platforms are fabricated via the solvent‐driven infiltration of polymer (SIP) technique [38, 39, 40, 41], which avoids thermal processing and thereby eliminates unintended thermal effects that could influence depolymerization behavior (Figure 4a). A bilayer structure is prepared by depositing an NP layer onto a PMMA layer, followed by exposure to high‐solvation solvent vapor (tetrahydrofuran; THF) to induce PMMA infiltration. The solvent vapor undergoes capillary condensation within the interstitial pores, and the condensed solvent plasticizes the underlying PMMA layer. Upon plasticization, the polymer dissolves into the condensed solvent and progressively diffuses into the interstitial pore network [38]. For the 75 and 22 nm NPs, ∼45 nm of PMMA film is infiltrated into ∼240 nm of NP packing, which possesses a solid volume fraction of ∼64% (corresponding to a porosity of ∼36 vol.%), resulting in ∼50% filling of the available pore volume. Since smaller NPs promote stronger capillary condensation due to the reduced pore size and higher surface curvature [20], the pores of the 7 nm NP packing are already partially occupied by capillary‐condensed water from ambient conditions. To account for the reduced accessible pore volume, a thinner PMMA layer (∼30 nm) is employed for the 7 nm NP system. As shown in the SEM image in Figure 4b (75 nm NP case), the interstitial voids between the NPs are filled with PMMA, confirming that the SIP technique enables effective polymer infiltration while preserving the disordered packing structure.

**FIGURE 4:**
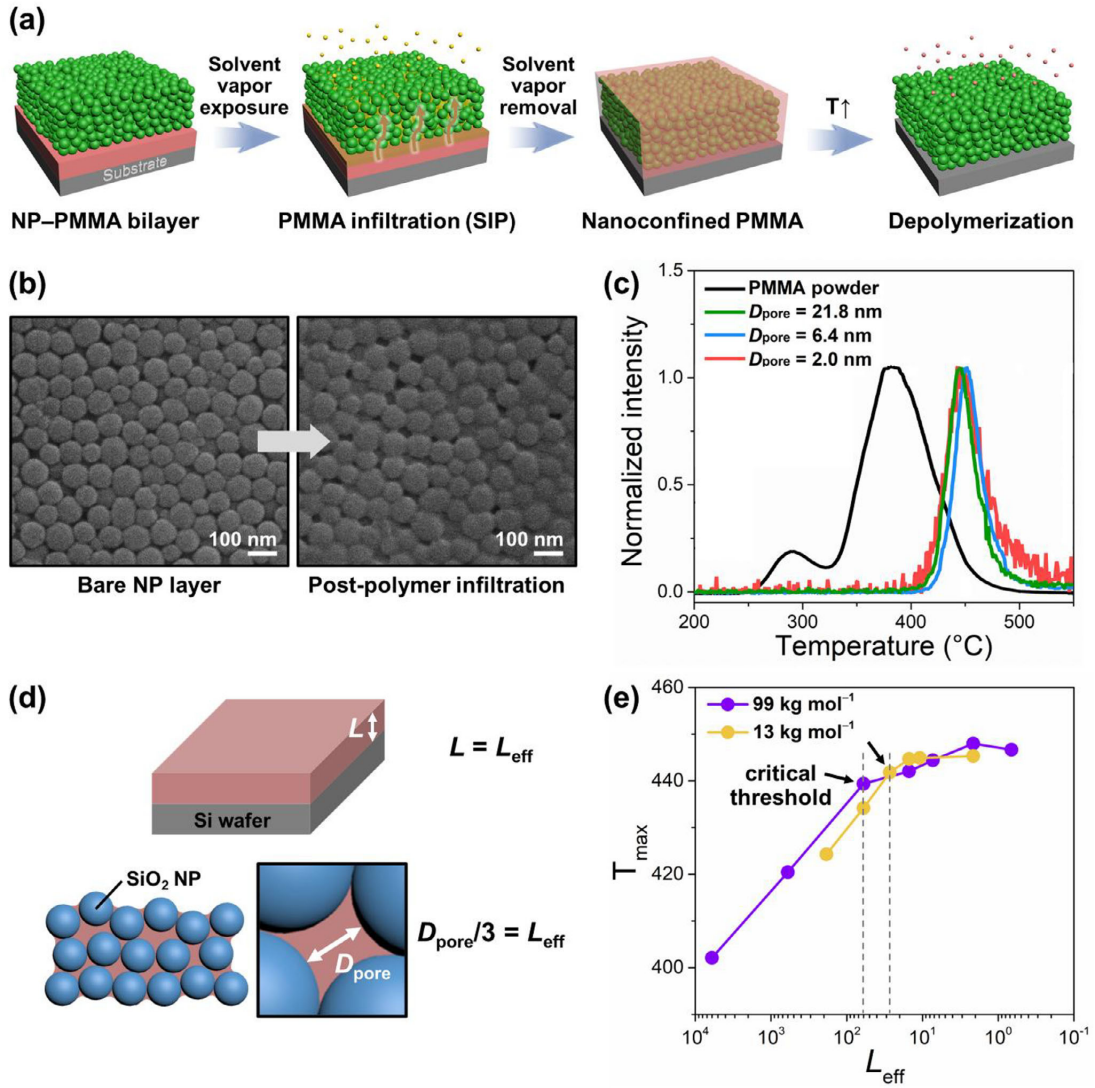
(a) Schematic illustration of the solvent‐driven infiltration of polymer (SIP) process for preparing nanocomposites and the subsequent thermal depolymerization of confined PMMA through heating. (b) Top‐view SEM images of the 75 nm NP–PMMA bilayer before and after polymer infiltration, confirming pore filling while preserving the disordered packing structure. (c) Normalized temperature‐dependent ion evolution profiles (m/z = 41) of PMMA nanoconfined within SiO_2_ NP packings of different diameters (*D*_NP_ = 75 nm, *D*_pore_ = ∼21.8 nm; *D*_NP_ = 22 nm, *D*_pore_ = ∼6.4 nm; and *D*_NP_ = 7 nm, *D*_pore_ = ∼2.0 nm), compared with bare PMMA powder. (d) Schematic definition of *L*_eff_ for thin films on planar SiO_2_ substrates and for polymers confined within interstitial pores of SiO_2_ NP packings. (e) *T*_max_ of PMMA with different molecular weights (*M*_n_ = 99 and 13 kg mol^−1^) plotted as a function of *L*_eff_, demonstrating a unified master curve across distinct confinement geometries.

The normalized ion evolution profiles for all three pore sizes (∼21.8, ∼6.4, and ∼2.0 nm) exhibit essentially identical shapes and peak positions, as shown in Figure 4c. Relative to the bulk state, the peak associated with chain‐end‐initiated unzipping is effectively suppressed; at the same time, the onset depolymerization temperature shifts by 150°C (from ∼250°C to ∼400°C), and the peak for random‐chain‐scission‐initiated unzipping moves to a significantly higher temperature with a *T*_max_ of ∼445°C. The invariance of the ion evolution profiles across this wide range of pore sizes demonstrates that once the confinement length falls below the critical threshold, further reductions in spatial dimension no longer affect depolymerization kinetics. In this regime of extreme nanoconfinement, segmental mobility is already maximally constrained by the dense SiO_2_ interfaces; consequently, additional decreases in pore size do not produce further increases in *T*_max_, indicating the onset of a confinement‐saturated stabilization regime.

To reconcile the 1D and 3D nanoconfinement environments, we introduce an effective confinement length (*L*_eff_) that places both geometries on a unified physical scale, as schematically illustrated in Figure 4d. The definition of *L*_eff_ is based on the characteristic length associated with the volume‐to‐surface ratio (*V*/*S*), which governs the relative influence of interfacial interactions. For thin films, the confinement length scales with the film thickness, *L*, since the *V*/*S* ratio calculated using only the confining substrate interface is proportional to *L*. For the 3D interstitial pore geometry, applying the same principle and directly calculating *V*/*S* for a random dense packing of spheres, we obtain *L*_eff_ = *D*_pore_/3 (see Note S1 for detailed derivation). When *T*_max_ values are plotted as a function of *L*_eff_ (Figure 4e), data obtained from both thin‐film and NP confinement geometries collapse onto single master curves (one for each molecular weight). This collapse demonstrates that depolymerization kinetics are governed by a universal confinement length scale that is independent of the specific confinement architecture.

The deviation observed in the thin‐film series between 60 and 15 nm thickness (Figure 3c) is consolidated within this unified representation, revealing a plateau below *L*_eff_ ∼60 nm. A similar trend is observed for the lower‐molecular‐weight PMMA (*M*_n_ = 13 kg mol^−1^), which exhibits a plateau below *L*_eff_ ∼27 nm, confirming that the scaling relationship is fundamentally independent of molecular weight. This unified scaling suggests that the apparent saturation observed in the NP‐confined systems represents the asymptotic limit of confinement‐induced thermal stabilization as *L*_eff_ approaches molecular length scales. The resulting master curve establishes *L*_eff_ as a universal descriptor governing thermal depolymerization kinetics across distinct nanoconfinement architectures, whether imposed by a planar interface or a 3D geometry of NP packings.

We note, however, that PMMA–SiO_2_ interactions are present as a consistent background condition throughout this study, and their contribution to the observed stabilization cannot be completely separated from geometric confinement in the present system. We further note that *L*_eff_, as defined here, is not a measure of pure geometric confinement alone; it is an effective length scale that reflects the combined influence of geometric confinement and interfacial interaction strength. Accordingly, *L*_eff_ should be interpreted as a unifying descriptor of the confinement‐driven stabilization behavior observed across architectures, rather than a purely geometric quantity such as pore size or film thickness. Future studies employing weakly interacting NP surfaces or alternative polymer–substrate combinations will be valuable for quantitatively decoupling the contributions of geometric confinement and polymer–surface interactions.

## 3 Conclusions

In summary, we show that radical depolymerization kinetics in polymers can be governed by nanoscale confinement geometry. Confinement suppresses chain‐end‐initiated unzipping and increases the effective energetic barrier for random chain scission, resulting in pronounced thermal stabilization. We attribute this behavior to restricted segmental mobility near polymer– SiO_2_ interfaces, which imposes diffusion‐related kinetic constraints on reactive radical species. A systematic investigation spanning 1D thin films (*L*, 15 nm to 6 µm) and 3D interstitial pores in NP packings (*D*_pore_, ∼2.0 to ∼21.8 nm) reveals that this stabilization is governed by a universal effective confinement length, *L*_eff_. When expressed in terms of *L*_eff_, depolymerization temperatures from both thin‐film and NP confinement geometries collapse onto a single master curve, identifying a critical threshold at *L*_eff_ ∼60 nm (∼7.2 *R*_g,0_ of the PMMA chains), consistent with previously reported confinement‐dominated regimes [42, 43]. Below this length scale, depolymerization enters a confinement‐saturated regime in which further reductions in spatial dimension no longer increase the depolymerization temperature. These findings establish nanoconfinement as a tunable physical parameter for modulating radical depolymerization pathways, providing a quantitative framework for optimizing monomer recovery in next‐generation circular polymer upcycling strategies and offering practical design guidance for catalytic depolymerization reactors. For example, pore or channel dimensions should be engineered above the critical confinement threshold (∼7 *R*_g,0_) to avoid unintentionally suppressing depolymerization kinetics.

Furthermore, the findings of this study have important implications for the design of thermally robust polymer nanocomposites with high NP loadings. The nanocomposite platform fabricated via the SIP technique in this work contains ∼64 vol.% of NPs, significantly exceeding the filler contents typical of conventional polymer nanocomposites [38, 39, 40, 41]. Such nanocomposites are known to exhibit exceptional mechanical and transport properties [41]. A previous study has shown that dense NP packings can substantially elevate the thermo‐oxidative decomposition threshold of polystyrene, attributed to restricted diffusion of free radicals, oxygen, and reaction products, which effectively increases the activation energy for degradation [29]. Combined with our observation of suppressed depolymerization under nanoconfinement, these results suggest that highly loaded polymer–NP nanocomposites exhibit enhanced resistance to both thermo‐oxidative degradation and thermal depolymerization. More broadly, these findings highlight nanoconfinement as a fundamental physical parameter that can be used to control radical reaction kinetics in polymeric materials.

## 4 Experimental Section

### 4.1 Materials

Monodisperse PMMA samples (P40228‐MMA, *M*_n_ = 99 kg mol^−1^, PDI = 1.12, Link: https://www.polymersource.ca/index.php?route = product/category&path = 2_2183_14_84_2188_2708_1242&product_id = 11362&subtract = 0&serachproduct = yes&categorystart = A‐1.1; and P40551‐MMA, *M*_n_ = 13 kg mol^−1^, PDI = 1.6, Link: https://www.polymersource.ca/index.php?route = product/category&path = 2_2183_14_84_2188_2708_1242&product_id = 11880&subtract = 1&serachproduct = yes&categorystart = A‐1.1), synthesized via anionic polymerization, are purchased from Polymer Source, Inc. (Dorval, QC, Canada). Toluene (HPLC grade) is purchased from Thermo Fisher Scientific (Pittsburgh, PA, USA). THF (≥99.0%), hydrochloric acid (ACS reagent, 37 wt.%), and aqueous dispersions of SiO_2_ NPs with diameters of 7 nm (Ludox SM‐30) and 22 nm (Ludox TM‐50) are purchased from Sigma–Aldrich (St. Louis, MO, USA). Aqueous dispersions of SiO_2_ NPs with diameters of 75 nm (Snowtex ST‐YL) and 112 nm (Snowtex ST‐ZL) are generously provided by Nissan Chemical America Corporation (Houston, TX, USA). GPTES (≥97%) is purchased from Gelest (Morrisville, PA, USA).

### 4.2 Preparation of PMMA Films

Prior to film deposition, Si wafers (452, UniversityWafer, South Boston, MA, USA) are cut into 1.5 × 1.5 cm^2^ squares, rinsed with isopropyl alcohol and deionized water, and subsequently dried with nitrogen. The wafers are treated with oxygen plasma (PDC‐32G, Harrick Plasma, Ithaca, NY, USA) for 5 min to eliminate any potential residual organic contaminants. The PMMA solutions are prepared by dissolving PMMA in toluene at various concentrations ranging from 1 to 6 wt.%. The PMMA films with thicknesses ranging from 15 to 600 nm are deposited onto the Si wafers by spin coating the PMMA solutions using a spin coater (WS‐400BZ‐6NPP/Lite spin coater, Laurell Technologies, North Wales, PA, USA), where the thickness is controlled by adjusting the solution concentration and rotation speed. Drop casting is employed for the thicker films of 6 and 60 µm, with the thickness controlled by adjusting the solution concentration. To moderately weaken the interactions between the PMMA and the substrate, a ∼10 nm‐thick Au layer is deposited on the Si wafers via magnetron sputtering (Explorer‐14, Denton Vacuum, Moorestown, NJ, USA).

For in situ ellipsometry measurements, Si wafer surfaces are modified with GPTES‐derived epoxy groups according to a previously reported procedure [50]. GPTES is employed to minimize interactions between the PMMA and the substrate. A 1:1 (v/v) mixture of water (28 mL) and ethanol (28 mL) is prepared, and the pH is adjusted to 4 using 1 M hydrochloric acid. Subsequently, GPTES (3 mL) is added to the solution. Si wafers (1.5 × 1.5 cm^2^) pre‐treated with oxygen plasma for 5 min are immersed in the mixture and gently stirred for 3 h at room temperature. The silanized wafers are then rinsed with water. Finally, the PMMA layer is deposited onto the modified substrates via spin coating without further oxygen plasma treatment.

### 4.3 Preparation of PMMA Nanoconfined Within NP Packings

The stock SiO_2_ NP aqueous dispersions are diluted with deionized water to appropriate concentrations and then sonicated for at least 2 h to ensure a homogeneous dispersion of the SiO_2_ NPs, followed by filtration using hydrophilic syringe filters with a size cutoff of 0.45 µm (09‐720‐005, Thermo Fisher Scientific) to remove potential NP agglomerates. Following the procedure described above, a PMMA layer is coated onto the wafer surface to a thickness of ∼45 nm for 112, 75, and 22 nm NP layers and ∼30 nm for 7 nm NP layers. Then, the PMMA film is oxygen‐plasma‐treated for 5 s to render the film surface hydrophilic; the NP layer with a thickness of ∼240 nm is then spin‐coated onto this surface to form a bilayer film. The thickness of each layer is controlled by changing rotation speed. The resulting bilayer film is exposed to THF vapor for 30 min to facilitate SIP, yielding the nanoconfined composite (see Figure S6 for details).

### 4.4 Characterization

The thermal depolymerization behavior is monitored by TGA–MS using a quadrupole mass spectrometer (Stanford Research Systems, RGA‐100). All TGA–MS measurements are performed under light‐shielded conditions to prevent exposure to ambient light, including ultraviolet irradiation, throughout the depolymerization process. The samples are pretreated to 100°C in a high vacuum of ∼10^−8^ Torr to remove any adsorbed species and residual solvent. Following this stage, the samples are heated from room temperature to 600°C at a heating rate of 10°C min^−1^ while the desorbing species are monitored. Given the sub‐micrometer sample thicknesses, high vacuum, and moderate heating rate, monomer diffusion out of the samples is expected to be much faster than the depolymerization reaction itself, minimizing its contribution to the measured onset and peak temperatures. The size and structure of the samples are examined using a field emission scanning electron microscope (FE‐SEM) (JSM‐7500F, JEOL, Japan).

The thickness and refractive index of PMMA films are monitored in situ using a spectroscopic ellipsometer (alpha‐SE, J. A. Woollam, Lincoln, NE, USA). The samples are mounted onto the temperature‐controlled stage (THMS350V, Linkam Scientific Instruments, Tadworth, UK) using thermal paste, and ellipsometry data are collected in the wavelength (*λ*) range of 380−900 nm at an incident angle of 70° during the heating cycles between 40°C and 350°C. The film samples are heated at a heating rate of 10°C min^−1^ to a set temperature of 350°C. The ellipsometer measures the amplitude ratio (*Ψ*) and the phase difference (*Δ*) of the complex reflection coefficients of light polarized parallel and perpendicular to the plane of incidence. The optical data obtained are analyzed using the CompleteEASE software to extract the thickness and refractive index. Each transparent layer is modelled as a Cauchy layer, and the refractive index is represented by the equation *n* = A + B/*λ* ^2^ + C/*λ* ^4^, where A, B, and C are optical constants and *λ* is the wavelength of light. Modelling is performed with a small mean square error (<10). The T_g_ and the onset temperatures for chain‐end‐initiated unzipping and random‐chain‐scission‐initiated unzipping are determined by the intersection of two linear slopes within the transition regimes of the thickness–temperature profiles.

## Conflicts of Interest

The authors declare no conflicts of interest.

## Supporting information




**Supporting File**: smll75295‐sup‐0001‐SuppMat.docx.

## Data Availability

The data that support the findings of this study are available from the corresponding author upon reasonable request.
