# Supplementary material for: Universal Confinement Length Governs Radical Depolymerization Kinetics in Nanoconfined Polymers
Source: Small. 2026 Aug 18;22(59):e75295. doi: 10.1002/smll.75295 (PMC13690613; doi:10.1002/smll.75295)
Supplement: Supplementary file 1 [file SMLL-22-e75295-s001.docx]

**Supporting Information**

**Universal Confinement Length Governs Radical Depolymerization Kinetics in Nanoconfined Polymers**

*Uiseok Hwang, Ching-Yu Wang, Zhanyuan Liu, John M. Vohs, Baekmin Q. Kim^*^, and Daeyeon Lee^*^*

Dr. U. Hwang, Dr. C.-Y. Wang, Z. Liu, Prof. J. M. Vohs, and Prof. D. Lee

Department of Chemical and Biomolecular Engineering, School of Engineering and Applied Science, University of Pennsylvania, Philadelphia, Pennsylvania 19104, United States

E-mail: daeyeon@seas.upenn.edu (Prof. D. Lee)

Prof. B. Q. Kim

Department of Battery and Chemical Engineering, Hanyang University ERICA, Ansan, Gyeonggi 15588, Republic of Korea

E-mail: [baekminkim@hanyang.ac.kr](mailto:baekminkim@hanyang.ac.kr) (Prof. B.Q. Kim)

**Note 1. Derivation of the effective confinement length (*L*_eff_) for the 3D interstitial pore geometry**

For the 1D thin-film geometry, *L*_eff_ is defined as the film thickness (*L*) itself. This follows from taking the volume-to-surface ratio using only the confining (interacting) surface—i.e., the substrate interface, excluding the free surface exposed to vacuum/air, which does not impose confinement. For a film of area (*A*) and thickness (*L*), this gives *L*_eff_ = *V*/*S* = (*A*·*L*)/*A* = *L*.

We validate the *L*_eff_ = *D*_pore_/3 approximation used in the main text by directly calculating the volume-to-surface ratio of a random dense packing of spherical NPs, applying the same principle. For the 3D interstitial pore geometry, the entire pore boundary consists of confining NP surface, so the total NP surface area is used as *S*.

**1. Setup**

Consider a random dense packing of monodisperse spherical SiO_2_ NPs with diamter (*D*_NP_) and solid volume fraction *Φ* ≈ 0.64 (random close packing). The pore volume fraction is therefore (1 − *Φ*) ≈ 0.36. For a single sphere of radius, *r* = *D*_NP_/2, the volume and surface area are:

*v*_sphere_ = 4πr^3^/3	(S1)

*s*_sphere_ = 4πr^2^	(S2)

**2. Surface area per unit volume of the packing**

Within an arbitrary unit volume of the packing (*V*_total_ = 1), the number of spheres (*N*) present is set by the solid volume fraction:

*N* = $\frac{\text{Φ} \text{V}_{\text{total}}}{\text{v}_{\text{sphere}}}$ = $\frac{\text{Φ}}{\text{4}\text{π}\text{r}^{\text{3}}\text{/3}}$	(S3)

The total surface area (*S*) contributed by these spheres per unit volume is then:

*S* = *N*·*s*_sphere_ = $\frac{\text{Φ}}{\text{4}\text{π}\text{r}^{\text{3}}\text{/3}}$·4πr^2^ = $\frac{\text{3}\text{Φ}}{\text{r}}$	(S4)

**3. Pore volume**

The geometric pore volume (*V*_pore_) is:

*V*_pore_ = (1 − *Φ*) *V*_total_	(S5)

**4. Effective confinement length (*L*_eff_)**

The effective confinement length is defined as the volume-to-surface ratio:

*L*_eff_ = $\frac{\text{V}_{\text{pore}}}{\text{S}}$ = $\frac{\text{(1 }\text{- }\text{Φ}\text{)}}{\text{6}\text{Φ}}$·*D*_NP_	(S6)

Evaluating the numerical prefactor using *Φ* ≈ 0.64:

*L*_eff_ = $\frac{\text{(1 }\text{- }\text{Φ}\text{)}}{\text{6}\text{Φ}}$·*D*_NP_ = $\frac{\text{(1 }\text{- }\text{0.64}\text{)}}{\text{6 × 0.64}}$·*D*_NP_ = 0.094·*D*_NP_	(S7)

**5. Conversion to the interstitial pore diameter (*D*_pore_)**

Using the known geometric relation between the interstitial pore diameter and the NP diameter for a random dense packing, *D*_pore_ = 0.29·*D*_NP_, L_eff_ can be re-expressed in terms of the experimentally reported pore diameter:

*L*_eff_ = 0.094·$\frac{\text{D}_{\text{pore}}\text{ }}{\text{0.29}}$ = 0.324·*D*_pore_ ≈ *D*_pore_/3	(S8)

**Note 2. Nanoconfinement effects in lower-molecular-weight PMMA**

The thermal depolymerization behavior of the lower-molecular-weight PMMA (M_n_ = 13 kg mol^−1^, PDI = 1.6) provides critical insight into molecular weight effect and compelling validation of our scaling model (Figure S3). Unlike the high-molecular-weight PMMA (M_n_ = 99 kg mol^−1^), which remains thermally stable until ~250 °C, the lower-molecular-weight sample exhibits an early onset of decomposition at a significantly lower temperature of ~110 °C (Figure 2). This discrepancy is fundamentally rooted in the higher concentration of chain ends inherent to shorter polymer chains. The increased density of these unstable initiation sites triggers chain-end-initiated unzipping at lower thermal energies; however, this process is effectively suppressed with decreasing *L*_eff_. At the same time, decreasing *L*_eff_ leads to a progressive increase in T_max_, consistent with the trend observed in the high-molecular-weight system.

**Note 3. Experimental details in SIP process**

The SIP process enables rapid infiltration of PMMA into interstitial pores of NPs via solvent-assisted solvation (Figure S6a). In particular, THF facilitates remarkably fast kinetics due to its strong hydrogen-bonding interactions with PMMA. However, excessive exposure to solvent vapor leads to structural degradation of the samples, as shown in Figure S6b. When analyzed via ellipsometry, these over-exposed samples exhibit high mean squared error values of >50, indicating significant inhomogeneity and optical anisotropy in the refractive index throughout the film. To balance infiltration efficiency and structural integrity, we identify 30 min as the optimal duration for achieving full infiltration without damaging the sample. This observation is further supported by optical microscopy (OM) analysis (Figure S6c), which reveals the formation of cracks and pinholes in samples subjected to prolonged solvent exposure.

This phenomenon occurs because the polymer chains preferentially migrate toward the sample surface, driven by the high thermodynamic affinity between the solvent and the polymer. Upon prolonged exposure to solvent vapor, the polymer is expelled toward the top interface, leading to subsequent dewetting of the film. As a definitive piece of evidence, we observe that infiltration does not occur in a polymer-on-top bilayer configuration, where the polymer is already positioned at the surface. This confirms that the driving force for structural change is the solvent-induced migration and eventual expulsion of the polymer chains to the exterior interface.

**Figure S1.** Temperature-dependent ion evolution profiles of PMMA powder showing characteristic fragment ions (m/z = 15, 39, 41, and 69).

**Figure S2.** Deconvoluted temperature-dependent ion evolution profile (m/z = 41) of PMMA powder. The two distinct peaks correspond to chain-end-initiated unzipping (8.7%) and random chain scission followed by unzipping (91.3%).

**Figure S3.** Data obtained from lower-molecular-weight PMMA (M_n_ = 13 kg mol^−1^, PDI = 1.6): normalized temperature-dependent ion evolution profiles (m/z = 41) of PMMA films with varying thicknesses on SiO_2_ surfaces (*L* = 185–15 nm) and PMMA nanoconfined within SiO_2_ NP packings of different diameters (*D*_NP_ = 112 nm, *D*_pore_ = ~32.5 nm, *L*_eff_ = ~10.8 nm; and *D*_NP_ = 22 nm, *D*_pore_ = ~6.4 nm, *L*_eff_ = ~2.1 nm), compared with bare PMMA powder.

**Figure S4.** Deconvoluted temperature-dependent ion evolution profile (m/z = 41) of a 6 μm-thick PMMA film on a SiO_2_ surface, showing two distinct peaks corresponding to chain-end-initiated unzipping and random chain scission followed by unzipping.

**Figure S5.** Deconvoluted temperature-dependent ion evolution profile (m/z = 41) of a 60 nm-thick PMMA film deposited on Au surface.

**Figure S6.** (a) Schematic illustration of the SIP process for the fabrication of PMMA–SiO_2_ nanocomposites. (b) Digital photograph of nanocomposite samples as a function of solvent-annealing time. (c) OM images of the nanocomposites at various solvent-annealing times shown at the same magnification.
